# Supplementary material for: Evaluating Mortality Response Associated with Two Different Nordic Heat Warning Systems in Riga, Latvia
Source: Int J Environ Res Public Health. 2020 Oct 22;17(21):7719. doi: 10.3390/ijerph17217719 (PMC7672594; doi:10.3390/ijerph17217719)
Supplement: Supplementary file 1 [file ijerph-17-07719-s001.pdf]

Supplementary Material

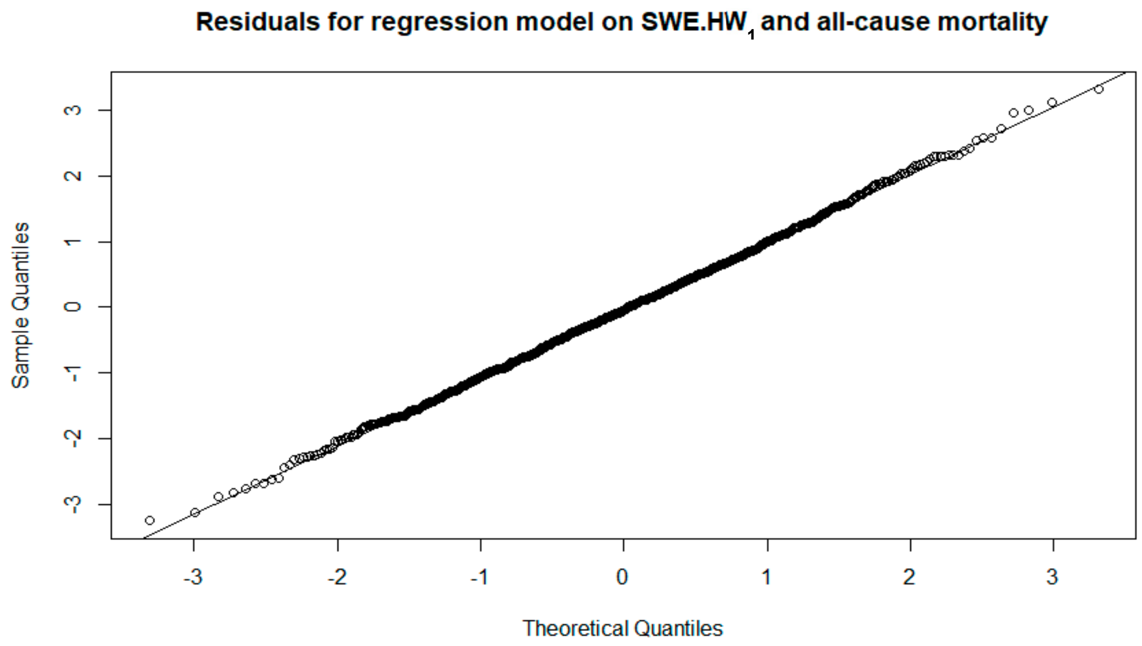

Figure S1. Scatter plot of the residuals for regression model on SWE.HW1 and all-cause mortality for Riga during the summer months 2009–2015.
